# High-quality metagenome-assembled genomes of bacteria associated with long-term cultivated giant coenocytic green alga *Bryopsis*

**DOI:** 10.1128/mra.00722-25

**Published:** 2025-08-27

**Authors:** Kanta K. Ochiai, Atsushi Toyoda, Takehiko Itoh, Gohta Goshima, Kazuma Uesaka

**Affiliations:** 1 Sugashima Marine Biological Laboratory, Graduate School of Science, Nagoya University 12965, Toba, Japan; 2 Comparative Genomics Laboratory, National Institute of Genetics 26359, Mishima, Shizuoka, Japan; 3 Advanced Genomics Center, National Institute of Genetics 26359, Mishima, Shizuoka, Japan; 4 School of Life Science and Technology, Institute of Science Tokyo 13290, Meguro-ku, Tokyo, Japan; 5 Centre for Gene Research, Nagoya University 12965, Nagoya, Japan; Montana State University, Bozeman, Montana, USA

**Keywords:** green algae, *Bryopsis*, metagenomic analysis, bacteria, metagenome-assembled genomes of bacteria

## Abstract

Seaweeds often associate with coexisting bacteria to support nutrient cycling and help defend against pathogenic microbes. Here, we report 38 closed circular and 12 fragmented metagenome-assembled genomes (MAGs) from bacteria tightly associated with the long-term cultivated coenocytic macroalga *Bryopsis* sp. KO-2023 from Sugashima Island, Japan.

## ANNOUNCEMENT

Some bacteria promote the growth of coexisting seaweed species (1–3). The giant coenocytic alga *Bryopsis* is no exception, with *Ruegeria* spp. reported to enhance its growth and morphogenesis (4). However, multiple coexisting bacterial strains with *Bryopsis* make it difficult to investigate the full range of associated communities. In this study, we reconstructed a comprehensive MAG set of *Bryopsis*-associated bacteria using ultra-deep PacBio and Illumina metagenomic sequencing.

Two *Bryopsis* sp. KO-2023 (male and female) were collected on 7 November 2019 from an outdoor tank (34°29′05.7″N, 136°52′31.0″E) supplied with seawater directly pumped from the sea in front of the Sugashima Marine Biological Laboratory (5). All DNA extraction methods, the kit used, and the library preparation and sequencing procedures for Illumina NovaSeq6000 and PacBio CLR platforms were described in a previous study (6). An additional PacBio HiFi library was prepared using the SMRTbell Prep Kit 3.0 (Pacific Biosciences, CA, USA), without fragmentation. Short fragments were removed by size selection using the BluePippin system (Sage Science, MA, USA). Sequencing was performed on the Revio system using its polymerase kit (Pacific Biosciences), and HiFi reads were generated from two Revio SMRT cells. In total, 194,393,442 150 × 2 Illumina reads (DRA016314, DRA019801, and DRA019802), 7,277,871 Pacbio CLR reads (DRA016315; N50 33.7 kb), and 314,446 HiFi reads (DRA019803; N50 16.2 kb) were obtained. All software was used with default parameters unless otherwise noted. The reads were quality-filtered and adapter-trimmed using Filtlong v0.2.1 and Fastp v0.23.2 (7). To remove host-derived reads, filtered reads were mapped to the *Bryopsis* sp. KO-2023 (GCA_030272585.1) using minimap2 v2.24-r1122 for long reads (8), and Bowtie2 v2.4.1 with the “-I 0 -X 2000” for short reads (9). The unmapped long reads were assembled using metaFlye v2.9.5 (10). As a result, 38 closed, complete circular assemblies (>1 Mb) were produced. The preprocessed short reads were assembled using MEGAHIT v1.2.9 (11) and were also used in hybrid assemblies with long reads using SPAdes v3.13.1 (12). The short-read and long-read-based metagenomic assemblies were binned with MetaBAT2 v2.15 (13) and MaxBin v2.2.7 (14), and dereplicated using DAS_Tool v1.1.5-0 (15). To enhance the contiguity of fragmented bins, short and long reads that strictly mapped to each bin were collected and iteratively re-assembled using Unicycler v0.4.8 (16). Re-assembled bins showing higher contamination values than their originals, as assessed by CheckM2 v1.0.1 (17), were discarded. Finally, species-level non-redundant bin sets satisfying both the Average Nucleotide Identity (ANI) species boundary of 0.95 and the CheckM2 quality metrics: >90% completeness and <6% contamination were selected using dRep v3.0.0 (18). The remaining 50 bins were subsequently polished using Racon v1.4.17 (19) and Pilon v1.24 (20) for two and three times, respectively. DFAST v.1.2.14 (21) was used for gene annotation of each genome.

**Fig 1:**
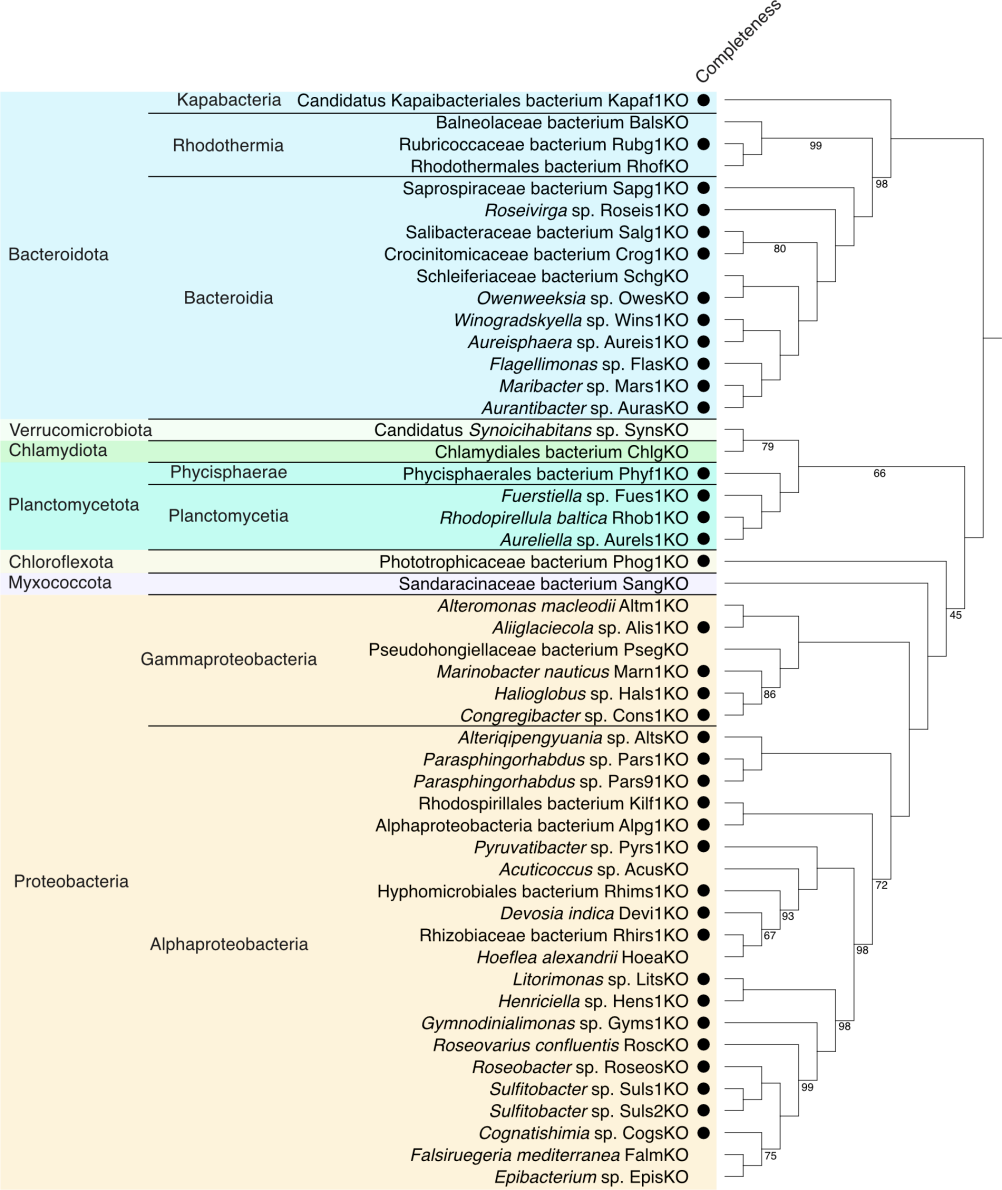
Taxonomic classification and phylogenetic relationships of bacterial MAGs reconstructed from *Bryopsis* sp. KO-2023. A phylogenetic tree of the 50 MAGs reconstructed in this study is shown. Closed circular MAGs are indicated with black circles. The maximum likelihood (ML) tree inference was performed based on 120 ubiquitous bacterial marker genes with GTDB-Tk v2.4.1 and the IQ-Tree2 package (23). The best-fit substitution model: “LG + F + R6” was selected using ModelFinder. Branch labels indicate bootstrap values estimated from ultrafast 1,000 bootstrap replicates; branches without labels have full bootstrap support.

The taxonomic classification of these bins, inferred by GTDB-Tk v2.4.1 with R220 database (22), is shown in Fig. 1 and Table 1. Of the 50 MAGs, 44 were considered to represent putative novel species based on the GTDB classification. This genome resource provides valuable insight into bacteria potentially involved in symbiosis with *Bryopsis*.

**TABLE 1:** Information of all MAGs

Name	GenBank accession numbers	Completeness (%) ^*a*^	Contamination (%) ^*a*^	N50 (bp)	Genome size (bp)	GC	Number of contigs	Relative sequencing depth (%)	MIMAG^*b*^	Number of rRNA genes
*Acuticoccus* sp. AcusKO	BAAHQC000000000.1	92.3	0.8	5,396,010	5430518	0.68	4	0.3	HG	5
Phototrophicaceae bacterium Phog1KO	AP041070.1	94.6	1.0	4,804,015	4804015	0.42	1	1.1	Fin.	6
*Aliiglaciecola* sp. Alis1KO	AP041047.1	100.0	0.0	4,667,729	4667729	0.43	1	0.3	Fin.	12
Alphaproteobacteria bacterium Alpg1KO	AP041048.1	97.9	0.7	4,035,276	4035276	0.57	1	0.3	Fin.	6
*Alteriqipengyuania* sp. AltsKO	AP041049.1	94.8	0.2	3,027,979	3027979	0.66	1	0.4	Fin.	3
*Alteromonas macleodii* Altm1KO	BAAHQG000000000.1	100.0	3.4	558,682	4656160	0.45	16	3.2	HG	15
*Aurantibacter* sp. AurasKO	AP041050.1	100.0	0.0	4630,471	4630471	0.40	1	0.2	Fin.	3
*Aureliella* sp. Aurels1KO	AP041052.1	99.3	1.7	7586122	7586122	0.54	1	0.4	Fin.	2
Balneolaceae bacterium BalsKO	BAAHQK000000000.1	98.1	0.8	3485342	3637937	0.37	4	0.4	HG	5
Chlamydiales bacterium ChlgKO	BAAHQL000000000.1	99.0	0.2	387379	1595787	0.43	9	1.8	HG	3
*Cognatishimia* sp. CogsKO	AP041053.1	94.5	0.2	3078439	3078439	0.55	1	1.3	Fin.	9
*Congregibacter* sp. Cons1KO	AP041054.1	100	0.0	3996987	3996987	0.58	1	0.8	Fin.	6
Crocinitomicaceae bacterium Crog1KO	AP041055.1	99.8	0.3	4102949	4102949	0.42	1	0.7	Fin.	6
*Devosia indica* Devi1KO	AP041056.1	99.9	0.1	4201733	4201733	0.62	1	0.3	Fin.	4
*Epibacterium* sp. EpisKO	BAAHQR000000000.1	100.0	1.4	662676	4426787	0.60	15	3.2	HG	15
*Maribacter* sp. Mars1KO	AP041065.1	100.0	1.0	5591320	5591320	0.42	1	0.6	Fin.	6
*Aureisphaera* sp. Aureis1KO	AP041051.1	99.7	0.1	3890200	3890200	0.41	1	0.8	Fin.	6
*Fuerstiella* sp. Fues1KO	AP041058.1	100.0	0.4	7906537	7906537	0.58	1	0.8	Fin.	2
*Halioglobus* sp. Hals1KO	AP041060.1	100.0	1.0	4739413	4739413	0.59	1	0.3	Fin.	6
*Henriciella* sp. Hens1KO	AP041061.1	100.0	0.1	3213697	3213697	0.53	1	0.8	Fin.	3
*Hoeflea alexandrii* HoeaKO	BAAHQY000000000.1	100.0	0.2	191722	4941777	0.62	32	1.2	HG	6
Candidatus Kapaibacteriales bacterium Kapaf1KO	AP041062.1	97.8	0.3	2975777	2975777	0.39	1	0.3	Fin.	3
Rhodospirillales bacterium Kilf1KO	AP041063.1	99.0	5.3	5797127	5797127	0.66	1	0.3	Fin.	6
*Litorimonas* sp. LitsKO	AP041064.1	100.0	0.1	3086194	3086194	0.58	1	24.5	Fin.	3
*Marinobacter nauticus* Marn1KO	AP041066.1	100.0	0.2	4008153	4008153	0.57	1	0.4	Fin.	9
*Flagellimonas* sp. FlasKO	AP041057.1	100.0	0.6	3867504	3867504	0.42	1	0.3	Fin.	6
Candidatus *Synoicihabitans* sp. SynsKO	BAAHRV000000000.1	96.9	0.6	76492	6040488	0.57	130	13.8	HG	3
*Parasphingorhabdus* sp. Pars91KO	AP041068.1	100.0	0.3	3412679	3412679	0.53	1	2.0	Fin.	3
*Parasphingorhabdus* sp. Pars1KO	AP041069.1	100.0	0.2	3643775	3643775	0.50	1	2.0	Fin.	3
Phycisphaerales bacterium Phyf1KO	AP041071.1	93.1	1.4	3198152	3198152	0.60	1	0.6	Fin.	3
Pseudohongiellaceae bacterium PsegKO	BAAHRI000000000.1	98.5	0.9	110680	4158577	0.57	65	0.2	MG	0
*Pyruvatibacter* sp. Pyrs1KO	AP041072.1	100.0	0.3	3439767	3439767	0.58	1	1.1	Fin.	3
Rhizobiaceae bacterium Rhirs1KO	AP041073.1	98.2	0.3	3806450	3806450	0.62	1	0.4	Fin.	3
Hyphomicrobiales bacterium Rhims1KO	AP041074.1	100.0	0.2	3750596	3750596	0.57	1	0.5	Fin.	3
*Rhodopirellula baltica* Rhob1KO	AP041075.1	100.0	3.1	7442493	7442493	0.55	1	0.3	Fin.	2
Rhodothermales bacterium RhofKO	BAAHRN000000000.1	97.3	0.0	69252	5655623	0.60	144	0.3	MG	1
*Gymnodinialimonas* sp. Gyms1KO	AP041059.1	99.9	0.1	4519048	4519048	0.63	1	3.9	Fin.	3
*Roseivirga* sp. Roseis1KO	AP041076.1	100.0	0.1	7760929	7760929	0.45	1	0.2	Fin.	6
*Roseobacter* sp. RoseosKO	AP041077.1	99.5	0.1	4461179	4461179	0.59	1	4.2	Fin.	3
*Roseovarius confluentis* RoscKO	AP041078.1	99.9	0.1	4318483	4318483	0.63	1	1.3	Fin.	4
Rubricoccaceae bacterium Rubg1KO	AP041079.1	95.5	0.0	4960933	4960933	0.65	1	0.5	Fin.	3
*Falsiruegeria mediterranea* FalmKO	BAAHQS000000000.1	100.0	0.1	253296	4968469	0.59	26	1.6	MG	0
Salibacteraceae bacterium Salg1KO	AP041080.1	98.7	1.8	3297375	3297375	0.44	1	0.1	Fin.	3
Sandaracinaceae bacterium SangKO	BAAHRS000000000.1	100.0	3.5	1970287	11686253	0.72	11	0.4	HG	3
Saprospiraceae bacterium Sapg1KO	AP041081.1	100.0	0.3	7323819	7323819	0.40	1	1.0	Fin.	6
*Owenweeksia* sp. OwesKO	AP041067.1	99.8	0.4	3395403	3395403	0.44	1	0.2	Fin.	3
Schleiferiaceae bacterium SchgKO	BAAHRU000000000.1	99.8	0.0	161439	2968640	0.44	25	0.2	MG	0
*Sulfitobacter* sp. Suls1KO	AP041082.1	97.8	0.1	4325308	4325308	0.61	1	3.9	Fin.	3
*Sulfitobacter* sp. Suls2KO	AP041083.1	100.0	0.1	3960145	3960145	0.60	1	16.1	Fin.	3
*Winogradskyella* sp. Wins1KO	AP041084.1	100.0	0.3	3603787	3603787	0.33	1	0.4	Fin.	6

^*a*^ Completeness and Contamination values were estimated using checkM2 (17).

^*b*^ HG: High-quality draft MAG. MG: Medium-quality draft MAG. Fin.: Finished MAG in MIMAG Consortium (24).

## Data Availability

MAG sequences and raw-read sequencing data are available under the NCBI project accessions PRJDB19662, PRJDB19663 and PRJDB15746. MAG sequences are also available on Figshare (10.6084 /m9.figshare.29334818). Accession numbers for each MAG are listed in Table 1. MAG re-assembly script is available at https://github.com/kazumaxneo/MAG_refinement.

## References

[1] Burgunter-Delamare B, KleinJan H, Frioux C, Fremy E, Wagner M, Corre E, Le Salver A, Leroux C, Leblanc C, Boyen C, Siegel A, Dittami SM. 2020. Metabolic complementarity between a brown alga and associated cultivable bacteria provide indications of beneficial interactions. Front Mar Sci 7:85. doi:10.3389/fmars.2020.00085

[2] Tapia JE, González B, Goulitquer S, Potin P, Correa JA. 2016. Microbiota influences morphology and reproduction of the brown alga Ectocarpus sp. Front Microbiol 7:197. doi:10.3389/fmicb.2016.00197 26941722 PMC4765120

[3] Wichard T. 2015. Exploring bacteria-induced growth and morphogenesis in the green macroalga order Ulvales (Chlorophyta). Front Plant Sci 6:86. doi:10.3389/fpls.2015.00086 25784916 PMC4347444

[4] Ochiai KK, Goshima G. 2025. Ruegeria strains promote growth and morphogenesis of the giant coenocytic alga Bryopsis. J Exp Bot:eraf262. doi:10.1093/jxb/eraf262 40515619 PMC12621095

[5] Shirae-Kurabayashi M, Edzuka T, Suzuki M, Goshima G. 2022. Cell tip growth underlies injury response of marine macroalgae. PLoS One 17:e0264827. doi:10.1371/journal.pone.0264827 35298494 PMC8929694

[6] Ochiai KK, Hanawa D, Ogawa HA, Tanaka H, Uesaka K, Edzuka T, Shirae-Kurabayashi M, Toyoda A, Itoh T, Goshima G. 2024. Genome sequence and cell biological toolbox of the highly regenerative, coenocytic green feather alga Bryopsis. Plant J 119:1091–1111. doi:10.1111/tpj.16764 38642374

[7] Chen S, Zhou Y, Chen Y, Gu J. 2018. fastp: an ultra-fast all-in-one FASTQ preprocessor. Bioinformatics 34:i884–i890. doi:10.1093/bioinformatics/bty560 30423086 PMC6129281

[8] Li H. 2018. Minimap2: pairwise alignment for nucleotide sequences. Bioinformatics 34:3094–3100. doi:10.1093/bioinformatics/bty191 29750242 PMC6137996

[9] Langmead B, Salzberg SL. 2012. Fast gapped-read alignment with Bowtie 2. Nat Methods 9:357–359. doi:10.1038/nmeth.1923 22388286 PMC3322381

[10] Kolmogorov M, Bickhart DM, Behsaz B, Gurevich A, Rayko M, Shin SB, Kuhn K, Yuan J, Polevikov E, Smith TPL, Pevzner PA. 2020. metaFlye: scalable long-read metagenome assembly using repeat graphs. Nat Methods 17:1103–1110. doi:10.1038/s41592-020-00971-x 33020656 PMC10699202

[11] Li D, Liu C-M, Luo R, Sadakane K, Lam T-W. 2015. MEGAHIT: an ultra-fast single-node solution for large and complex metagenomics assembly via succinct de Bruijn graph. Bioinformatics 31:1674–1676. doi:10.1093/bioinformatics/btv033 25609793

[12] Bankevich A, Nurk S, Antipov D, Gurevich AA, Dvorkin M, Kulikov AS, Lesin VM, Nikolenko SI, Pham S, Prjibelski AD, Pyshkin AV, Sirotkin AV, Vyahhi N, Tesler G, Alekseyev MA, Pevzner PA. 2012. SPAdes: a new genome assembly algorithm and its applications to single-cell sequencing. J Comput Biol 19:455–477. doi:10.1089/cmb.2012.0021 22506599 PMC3342519

[13] Kang DD, Li F, Kirton E, Thomas A, Egan R, An H, Wang Z. 2019. MetaBAT 2: an adaptive binning algorithm for robust and efficient genome reconstruction from metagenome assemblies. PeerJ 7:e7359. doi:10.7717/peerj.7359 31388474 PMC6662567

[14] Wu Y-W, Simmons BA, Singer SW. 2016. MaxBin 2.0: an automated binning algorithm to recover genomes from multiple metagenomic datasets. Bioinformatics 32:605–607. doi:10.1093/bioinformatics/btv638 26515820

[15] Sieber CMK, Probst AJ, Sharrar A, Thomas BC, Hess M, Tringe SG, Banfield JF. 2018. Recovery of genomes from metagenomes via a dereplication, aggregation and scoring strategy. Nat Microbiol 3:836–843. doi:10.1038/s41564-018-0171-1 29807988 PMC6786971

[16] Wick RR, Judd LM, Gorrie CL, Holt KE. 2017. Unicycler: resolving bacterial genome assemblies from short and long sequencing reads. PLoS Comput Biol 13:e1005595. doi:10.1371/journal.pcbi.1005595 28594827 PMC5481147

[17] Chklovski A, Parks DH, Woodcroft BJ, Tyson GW. 2023. CheckM2: a rapid, scalable and accurate tool for assessing microbial genome quality using machine learning. Nat Methods 20:1203–1212. doi:10.1038/s41592-023-01940-w 37500759

[18] Olm MR, Brown CT, Brooks B, Banfield JF. 2017. dRep: a tool for fast and accurate genomic comparisons that enables improved genome recovery from metagenomes through de-replication. ISME J 11:2864–2868. doi:10.1038/ismej.2017.126 28742071 PMC5702732

[19] Vaser R, Sović I, Nagarajan N, Šikić M. 2017. Fast and accurate de novo genome assembly from long uncorrected reads. Genome Res 27:737–746. doi:10.1101/gr.214270.116 28100585 PMC5411768

[20] Walker BJ, Abeel T, Shea T, Priest M, Abouelliel A, Sakthikumar S, Cuomo CA, Zeng Q, Wortman J, Young SK, Earl AM. 2014. Pilon: an integrated tool for comprehensive microbial variant detection and genome assembly improvement. PLoS One 9:e112963. doi:10.1371/journal.pone.0112963 25409509 PMC4237348

[21] Tanizawa Y, Fujisawa T, Nakamura Y. 2018. DFAST: a flexible prokaryotic genome annotation pipeline for faster genome publication. Bioinformatics 34:1037–1039. doi:10.1093/bioinformatics/btx713 29106469 PMC5860143

[22] Chaumeil P-A, Mussig AJ, Hugenholtz P, Parks DH. 2022. GTDB-Tk v2: memory friendly classification with the genome taxonomy database. Bioinformatics 38:5315–5316. doi:10.1093/bioinformatics/btac672 36218463 PMC9710552

[23] Nguyen L-T, Schmidt HA, von Haeseler A, Minh BQ. 2015. IQ-TREE: a fast and effective stochastic algorithm for estimating maximum-likelihood phylogenies. Mol Biol Evol 32:268–274. doi:10.1093/molbev/msu300 25371430 PMC4271533

[24] Bowers RM, Kyrpides NC, Stepanauskas R, Harmon-Smith M, Doud D, Reddy TBK, Schulz F, Jarett J, Rivers AR, Eloe-Fadrosh EA, et al.. 2017. Minimum information about a single amplified genome (MISAG) and a metagenome-assembled genome (MIMAG) of bacteria and archaea. Nat Biotechnol 35:725–731. doi:10.1038/nbt.3893 28787424 PMC6436528

